# Bailing out the bowel and bladder – A rare case report of rectal impalement injury with iatrogenic bladder rupture

**DOI:** 10.1016/j.ijscr.2021.105740

**Published:** 2021-03-11

**Authors:** Ganesan Balamurugan, Shirish Bhagvat, Amol Wagh, Hemant Jawale, Keerthika Reddy, Saurabh Jain

**Affiliations:** Department of General Surgery, Grant Medical College and Sir JJ Group of Hospitals, Byculla, Mumbai, 400008, India

**Keywords:** ICU, intensive care unit, IV, intravenous, POD, post operative day, OR, operating room, CT, computed tomography, PR, primary repair, DC, diversion colostomy, Impalement injury, Rectal perforation, Rectal foreign body, Colorectal injury, Bladder injury, Case report

## Abstract

**Introduction and importance:**

Rectal perforations due to foreign body impalement are infrequently encountered in practice. Accidental or intentional foreign body insertions pose a diagnostic challenge to surgeons and put them in demanding circumstances for successful extraction.

**Case presentation:**

We report a case of a 60-year-old male with alleged history of accidental foreign body insertion into the rectum. Radiographs showed a linear metallic foreign body with crooked end. Computed Tomography (CT) with rectal contrast revealed contrast extravasation indicating anterior wall perforation of upper rectum. Patient was taken urgently for exploration and foreign body removal. Intra-operatively, bladder was injured inadvertently. Primary repair of enterotomy and loop sigmoid-ostomy was done besides bladder repair. Patient tested positive for COVID-19. Patient was observed in critical care unit for two days. Post-operative period was unremarkable apart from midline abdominal wound gape for which secondary closure was done. Patient was discharged with urinary catheter in-situ. Patient followed-up with a normal cystourethrogram and a well-functioning stoma. Stoma closure after two months was uneventful.

**Clinical discussion:**

A thorough history and clinical examination is required and one should raise a high index of suspicion of perforation in patients with rectal foreign bodies, which should be managed appropriately and promptly to prevent sepsis and multi-organ dysfunction. Inadvertent bladder injuries are common with lower-midline incisions. However, give good results when repaired suitably.

**Conclusion:**

The rationale behind this report is to explicate the complexity and hurdles in the surgical management of rectal foreign bodies causing impalement injury.

## Highlights

• Rectal impalement injuries are infrequently encountered in surgeons’ practice. They pose a diagnostic challenge to surgeons and put them in demanding circumstances for successful extraction.
• High index of suspicion of perforations should be there in all rectal foreign bodies. Iatrogenic bladder injuries give excellent results when repair suitably.
• Prompt diagnosis and immediate intervention to remove the foreign bodies are quintessential in all rectal foreign bodies with evidence of perforation.

## 1 Introduction

The work has been reported in line with SCARE criteria [1]. Rectal impalement injury is an uncommon presentation to the emergency surgical units. Earliest reported cases of foreign body insertion causing anorectal trauma are dated in 1500s [2]. Insertion of rectal foreign bodies could be voluntary or accidental, related with sexual or non-sexual activities [3]. Extraction is always mandatory either by trans-anal route or trans-abdominal route bearing in mind the local and systemic complications. The final decision taken by the operating surgeon depending upon the size, shape, nature of the foreign body, mechanism of insertion and its exact site. Initial resuscitation and immediate removal reduce the incidence of morbidity and mortality in anorectal foreign bodies.

## 2 Presentation of case

A 60-year-old male presented with alleged history of inability to remove the accidentally lodged foreign body in the rectum. Patient gave history of self-administration of enema in the bathroom earlier, following which he slipped and landed on a bucket which had a long linear metallic rod with a crooked end standing upright. After failed attempts of removing the foreign body manually, patient developed pain in the lower abdomen and anal region which gradually progressed and forced the patient to seek medical care. Past history was unremarkable apart from chronic intermittent constipation and an open surgery done for left ureteric calculus 31 years back.

**Fig. 1:**
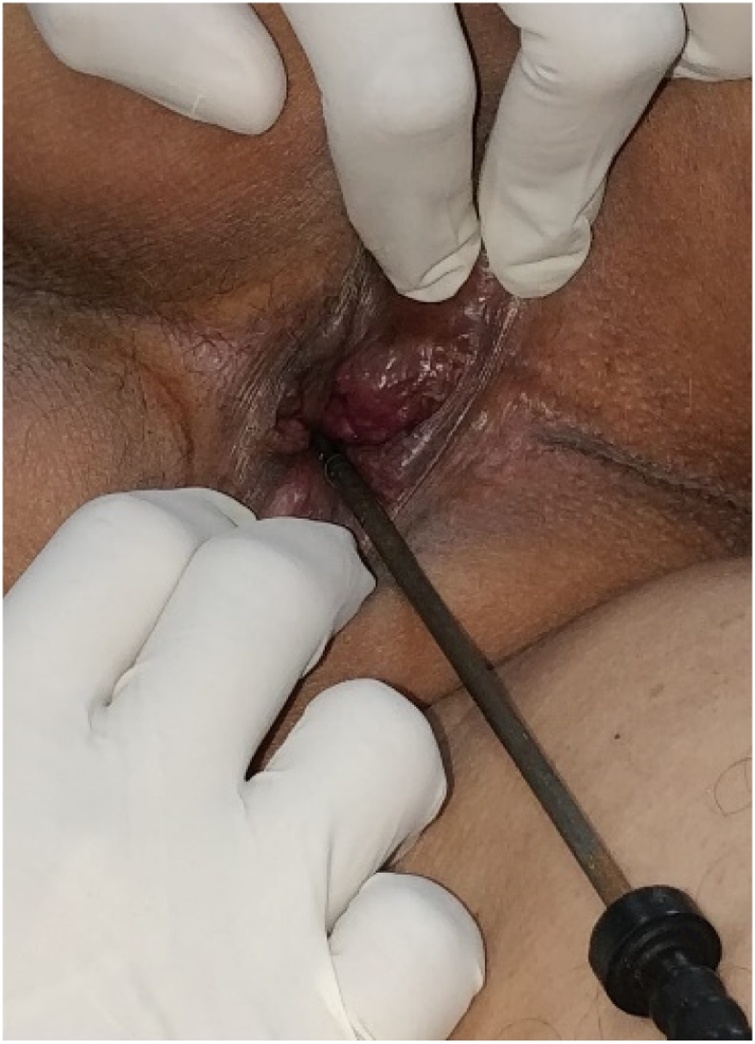
Shows the hanging metallic foreign body through the anus.

**Fig. 2:**
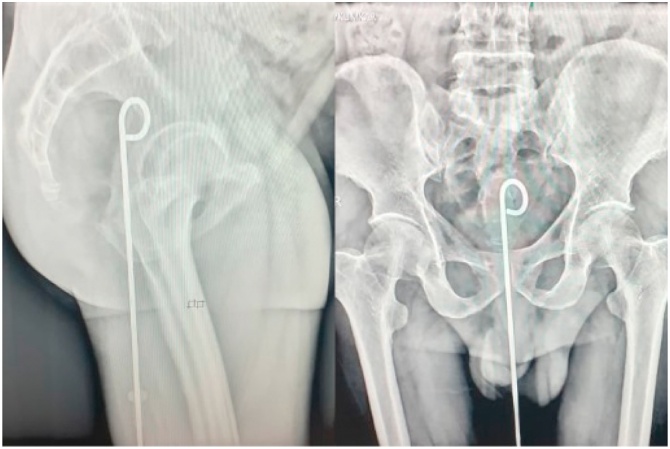
Represents the pelvic radiograph showing the metallic foreign body with crooked end.

Patient was hemodynamically stable. There was mild tenderness present in the lower quadrants of the abdomen and a metallic foreign body was found hanging out through the anus. Per rectal examination (Fig. 1) confirmed that there were no external and anal injuries and the upper end of the foreign body was not palpable. Pelvic radiography (Fig. 2) confirmed a foreign body with a crooked end in front of the rectal air-shadow signifying a potential rectal perforation. Patient was resuscitated immediately with intravenous (IV) crystalloids and broad-spectrum antibiotics. Rectal contrast enhanced CT revealed long linear metallic foreign body with beam hardening artefacts in lumen of anal canal, lower, mid and upper rectum with extravasation of contrast near upper rectum at the site of the crooked end of the foreign body with adjacent mild meso-rectal fat stranding suggestive of complete focal breach of upper anterior rectal wall.

Manipulation and trans-anal removal attempts were avoided taking into account the crooked end perforated the upper rectum which might cause further injury. Blood investigations were within normal limits. The patient and relatives were explained about the complexity of surgical intervention needed to remove the foreign body and stoma formation, if needed.

**Fig. 3:**
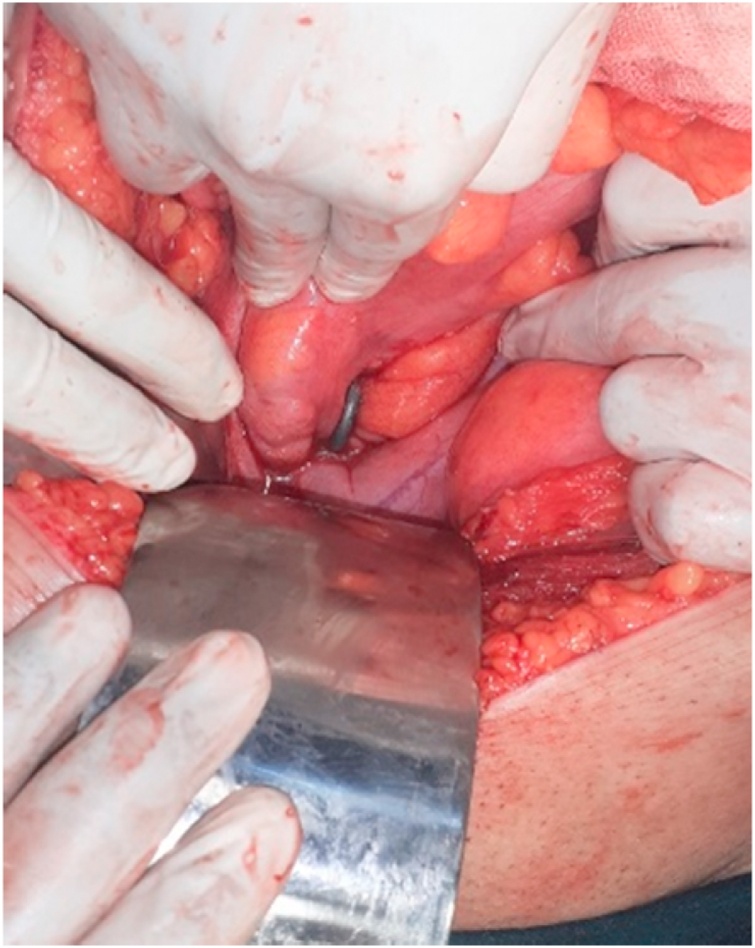
Shows the anterior wall perforation of the upper rectum by the foreign body.

**Fig. 4:**
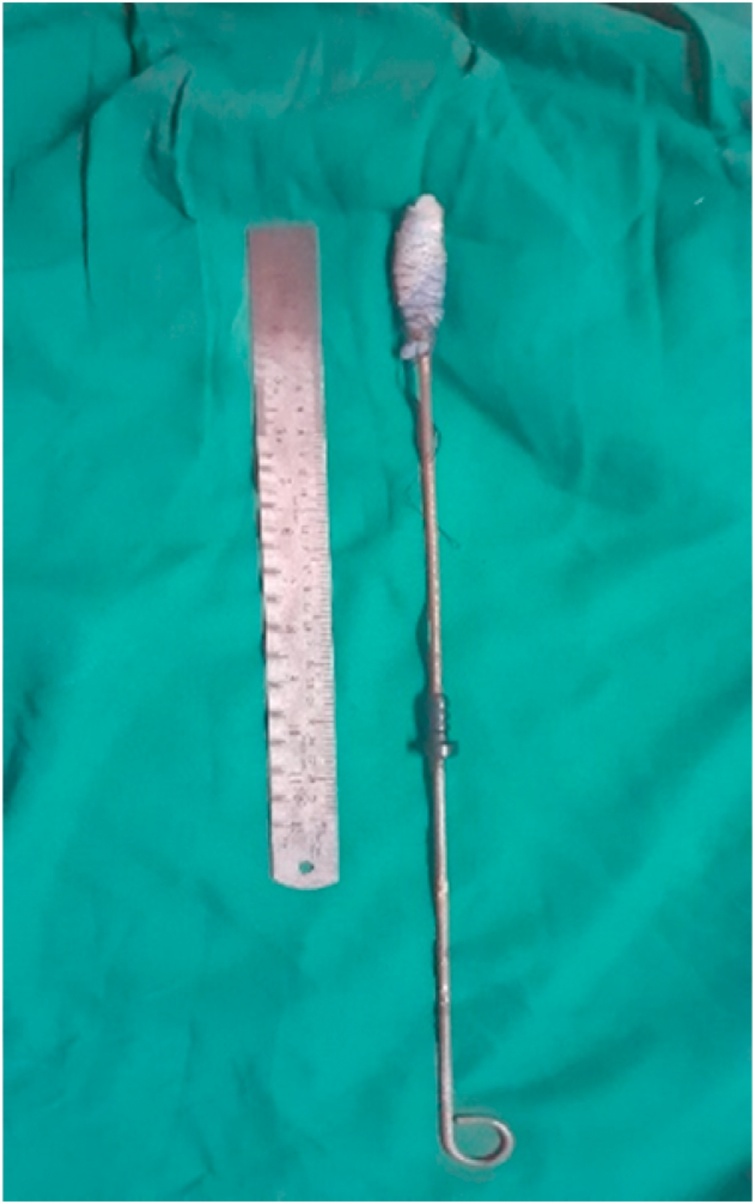
Shows the extracted foreign body.

Patient was taken urgently for Exploratory laparotomy. While exploring the pelvis the bladder was cut inadvertently with monopolar diathermy. Entire small bowel and large bowel was examined meticulously. On examining the rectum, it was found that the crooked end had perforated the anterior wall and was residing outside it, with the rest of the object inside the lumen. (Fig. 3). Repeated attempts in removal by the patient and the anatomy of rectum contributed to this complex lodgement of the foreign body. Because of this complexity, trans-anal removal was not possible without enterotomy. A 3 cm vertical enterotomy was made over the anterior wall of upper rectum in continuum with the perforation. Subsequently the foreign body was manipulated trans-anally to dislodge the crooked end and to facilitate its removal (Fig. 4) with an assistant pulling it out via anus. Extracted foreign body measured 46 cm in length. Enterotomy closed in two layers with simple interrupted sutures using silk 3′0. Bladder inner mucosa approximated using polyglactin 3′0 and seromuscular sutures using silk 3′0. Although there was no gross contamination, in order to prevent complications like post-op leak and also considering the concomitant bladder repair, a sigmoid colostomy was done.

Patient’s rt-PCR result came positive for COVID-19 post-operatively and was shifted to dedicated COVID-19 care centre and monitored in Intensive Care Unit (ICU) for 2 days. Enteral feeds started on Post-operative day (POD) 2. Stoma started functioning from POD 2. The repeat COVID-19 swab came negative after 5 days. A wound gape developed on POD 9 for which secondary suturing done on POD 21. Patient was taught stoma care and advised to keep urinary catheter in-situ for 3 weeks. Sutures were removed after 14 days. Patient reviewed with normal micturating cystourethrogram after 3 weeks showing normal bladder drainage with no extravasation or communication with rectum. Hence, the urinary catheter was removed.

**Fig. 5:**
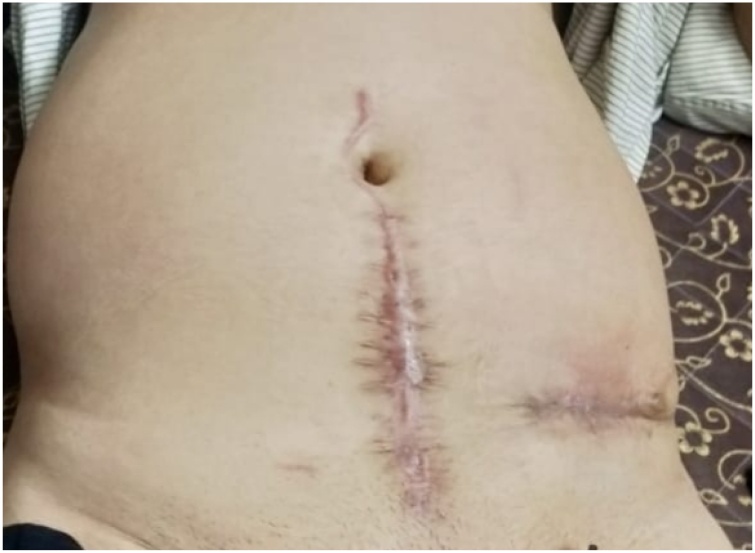
Depicts the patient’s abdomen after complete recovery.

Patient followed up for stoma closure after 8 weeks. Closure of loop sigmoid-ostomy was done with no complications. Post-operative period was uneventful. Patient passed flatus and stools on POD2. Patient was discharged on POD 5. On following up, patient is leading a normal life with unaltered bladder and bowel habits (Fig. 5).

## 3 Discussion

Rectal impalement encompasses foreign body trauma to the anus or rectum resulting in intra- peritoneal and extra-peritoneal injuries [4]. It occurs when a solid and elongated object pierces (entering in them mostly via anatomical orifices) and remains in the human body [5]. The incidence is more common in men. The most widespread form of introduction is through the anus. Rarely, orally ingested foreign bodies migrate towards the rectum and might cause injury.

Traditionally, impalement injuries are divided into three types: type I injuries that occur when a person in motion bums against an immobile object, type II when a mobile object collides with an immobile person and type III that results from an impact between a mobile object and a mobile person [6]. The present case is type I impalement injury (Table 1).

**Table 1:** Illustrates various categories of rectal foreign bodies. (our case comes under involuntary, non-sexual category which is rare).

	Voluntary	Involuntary
Sexual	• Autoeroticism (dildos, vibrator, etc) • Psychiatric illness	• Rape • sexual assault
Non-sexual	• Body packing of illegal drugs (packed in plastic bags, condoms, capsules, etc) • Attention seeking behaviour • Constipation relief (enema tips, toothpick, plastic bags, etc)	• Accidental (in children, elderly, mentally ill - toys, coins, erasers, thermometers, etc)

All rectal foreign bodies should be considered as potentially hazardous. Comprehensive history-taking, detailed information about the mechanism of foreign body ingestion/insertion and systematic clinical examination is mandatory to delineate the nature of foreign body and possible complications associated with it. In our case, the patient presented with mild pain in the anal region with a foreign body found hanging through the anus.

The most important step in the management of rectal foreign bodies is detecting whether there is perforation or not. By far, all the algorithms suggest that in case of perforation or peritonitis, patient should be taken to Operating Room (OR) immediately for either laparotomy or diagnostic laparoscopy. Many extraction techniques are available depending on the location of foreign body. Low lying foreign bodies can be attempted to evacuate per-anally in the emergency room itself. After excluding the perforation, High lying foreign bodies can be extracted under vision with adequate analgesia or conscious sedation either manual manipulation or with surgical instruments present in the emergency room [7].

Kyle et al. (2012) proposed an algorithm which suggested all failed cases after trans-anal or endoscopic extraction methods should undergo either mini-laparotomy/laparoscopy with direct pressure or laparotomy regardless the presence or absence of signs of peritonitis [7].

In our case, the patient’s CT confirmed anterior wall-upper rectal perforation. Hence patient was taken up for surgery without delay. Although, the complex nature of the foreign body and the mechanism of insertion, the foreign body did not cause any sphincter injury, intra-peritoneal or extraperitoneal damage. Urinary bladder is the most frequently injured organ during pelvic surgeries [8]. Prompt identification and immediate repair gives excellent results. Continuous suspicion and alertness by the surgeon are crucial.

Primary Repair (PR) with or without anastomosis of all colorectal injuries should be attempted initially unless the colonic tissue itself is inappropriate for repair due to severe oedema or ischemia. Even though primary repair was done in our case, considering the age, iatrogenic bladder injury and to avoid the complications like post-operative leak, Diversion Colostomy (DC) was done which was successfully restored after 2 months. Despite abundant reports on anorectal foreign bodies, there have been no cases in the literature authenticating a distinctive instance such as ours, i.e., an elderly male with COVID-19 positive status who sustained an accidental rectal impalement injury leading to an anterior rectal perforation without any other major structural injury by one of the longest foreign bodies reported.

## 4 Conclusion

Impalement injury presenting with rectal foreign body is a rare experience for surgeons. Our case-report is a very unique and a rarity in routine practice owing to the complexity of the nature, dimensions of foreign body and the mechanism of insertion. Judicious fluid resuscitation and instantaneous surgical intervention is quintessential in all rectal foreign bodies with evidence of perforation. Surgeons should approach the lower midline incisions in a cautious manner. All cases of colorectal injuries, iatrogenic bladder injuries should be attempted with primary repair, provided there is no gross contamination, presence of tissue oedema/ ischemia or other unfavourable factors. With the aforesaid means, the fatality rate in rectal foreign bodies and impalement injuries is maximally reduced and the recovery rate hits almost hundred percent.

## Conflicts of interest

None.

## Sources of funding

None.

## Funding

This research did not receive any specific grant from funding agencies in the public, commercial, or not-for-profit sectors.

## Ethical approval

Not applicable.

## Consent

Written informed consent was obtained from the patient for publication of this case report and accompanying images. A copy of the written consent is available for review by the Editor-in-Chief of this journal on request.

## Author contribution

Dr. Shirish Bhagvat: Study design, Performed the surgery

Dr. Amol Wagh: Study design, Assisted for surgery

Dr. Saurabh Jain: Study design, Assisted for surgery

Dr. Hemant Jawale: Assisted for surgery, Data collection

Dr. Keerthika Reddy: Assisted for surgery, Data collection

Dr. Balamurugan Ganesan: Assisted for surgery, Data collection, Writing the paper

## Research registration

Not applicable.

## Guarantor

Balamurugan Ganesan.

## Provenance and peer review

Not commissioned, externally peer-reviewed.
